# Can Incentives Improve Population Health? An Interview With Bridget Booske, PhD

**Published:** 2010-08-15

Bridget Booske is the project director of *County Health Rankings* and deputy director of its parent project, Mobilizing Action Toward Community Health (MATCH), funded by the Robert Wood Johnson Foundation. She also directs the Making Wisconsin the Healthiest State project, a research and translation effort funded by the Wisconsin Partnership Program, University of Wisconsin School of Medicine and Public Health. In this interview, Dr. Booske gives an overview of the MATCH articles in the September 2010 issue of *Preventing Chronic Disease *and talks briefly about the role of incentives in public health.

**Figure:**
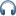
Can Incentives Improve Population Health? (MP3–8Mb)

00:00:00 Fran Kritz I’m Fran Kritz, editor of the Robert Wood Johnson Foundation Public Health Page. In 2009, the MATCH project was launched, Mobilizing Action Toward Community Health. We’re speaking today with Bridget Booske, who’s the deputy director of the MATCH project.
00:00:15 Kritz The MATCH project is funded by the Robert Wood Johnson Foundation. Bridget, thanks for being with us today.
00:00:22 Dr. Bridget Booske Thanks for having me.
Kritz We’re talking today because Preventing Chronic Disease, a journal of the Centers for Disease Control and Prevention, has published in this issue a set of essays that were commissioned by the MATCH project about improving population health.
00:00:39 Kritz Tell us about the essays in this issue of Preventing Chronic Disease.
Booske Dave Kindig, who is the co-director of the MATCH project, recruited a number of very interesting individuals from a variety of fields and application areas to write essays for us.
00:00:59 Booske John Witte wrote about experience and use of incentives in education. John Mullahy and Robert Haveman each came at it from an economic perspective. Dan Fox and David Asch and Rachel Werner talked about lessons that can be learned from health care. Ray Baxter talked about using existing programs and policies as incentives by making better use of regulations already in place. Tom Oliver talked about the use of rankings, and Peter Smith talked about the use of targets as incentives in Europe.
00:01:36 Kritz Let’s talk about incentives for a moment because that was the focus of some of the essays in this issue. What kinds of ideas do some of the authors present with respect to incentives for improving community health?
00:01:50 Booske Well, often when you think about incentives, people think about financial incentives — either rewarding certain kinds of performance or behaviors or penalizing poor performance, for example. But there are also a wide range of nonfinancial incentives that some of these essays discuss — things like regulations and legal ways to motivate health improvement. There are educational incentives as well, so there’s a wide range of different kinds of both financial and nonfinancial incentives that might improve population health.
00:02:31 Kritz Are there things that don’t work with respect to incentives when it comes to population health? Do you think?
00:02:35 Booske Sure, we can learn things from other fields such as the field of education, where various kinds of incentives have been in place. There we’ve learned that there’s a tendency for teachers, for example, when they have their performance being rewarded for good test scores for their students, there’s a tendency for them to teach towards the test. And so rather than teaching the broad curriculum, they will just teach materials specifically in the areas that they know they’re going to get tested on. There’s also some other things that can happen with incentives that have it not work right — gaming strategies or adverse selection or incentives just sometimes don’t always produce the desired outcome. If they’re not carefully designed, then they won’t work well.
00:03:29 Kritz People who have heard about incentives particularly for health know some standards — coffee shop gift cards and a slight reduction in a gym membership. But you’ve learned about some very unusual incentives as well. Can you give us an example?.
00:03:47 Booske Sure. There’s one quite interesting example from my home state of Wisconsin, where there’s a project that’s called Road Crew where young men between the ages of 21 and 34 who are most susceptible to drunk driving to both causing accidents and being injured in them and injuring others. This program called Road Crew involves providing these young men with limousine rides to and between and home from taverns. So they go out for an entire evening using a limo and at the end of the evening, they don’t have to admit to their friends that they’re too drunk to drive. They just say, “Oh, my limo is here. I’m leaving.” And this has proven to reduce drunk driving in the communities where this program has been implemented. And furthermore, it started off as a funded program but it’s now a fee-based system, so it’s self-sustaining. So the benefit to the participants must outweigh the cost in their minds, and so it’s a very successful program.
00:04:55 Kritz When it comes to the MATCH project, when you’ve looked at the various types of incentives, is it necessarily a prize or money or are there some unique incentives that might work for improving community health?
00:05:08 Booske Sure, as well as the financial incentives, and I talked about a few nonfinancial incentives. One that I didn’t mention was the whole concept of reputation as a motivational factor. And that’s what we are using in the MATCH project when we release the County Health Rankings. They, the notion of a poorer ranking is very motivating for communities to take action and try to figure out what they can do to improve their health and improve their rank and then ultimately their health.
00:05:47 Kritz Bridget, last question for you and that is, is there a timeline for beginning to look at some of the incentives and how well some of them work so that other communities might be able to implement those as well?
00:05:56 Booske Well, this is an ongoing area of investigation for us on the MATCH project. We are about halfway through our project and commissioning these essays and having experts such as Michael Rothschild, who wrote the social marketing essay talking about the Road Crew, and then people like Thomas Oliver, who is helping us think through how rankings can motivate performance — these people are helping us out with this research. And that we hope that over the next couple of years, we will be able to do more of pulling together, more information about what works and what doesn’t work for incentives.
00:06:37 Kritz The essays are in the September issue of Preventing Chronic Disease, a Centers for Disease Control and Prevention journal. Bridget Booske, deputy director of the MATCH project at the University of Wisconsin Population Health Institute, thanks so much for talking to us about this special issue.
00:06:53 Booske Thank you for having me. 
00:06:55 Kritz I’m Fran Kritz for the Robert Wood Johnson Foundation.

